# The development of recommendations for the assessment and management of sesamoiditis by podiatrists: A Delphi and content validity study

**DOI:** 10.1002/jfa2.12025

**Published:** 2024-05-31

**Authors:** Sarah Stewart, Preeti Kaur, Peta Tehan, Prue Molyneux, Matthew Carroll

**Affiliations:** ^1^ Department of Podiatry Auckland University of Technology Auckland New Zealand; ^2^ Auckland Sports Podiatry Auckland New Zealand; ^3^ School of Clinical Sciences Monash University Melbourne Victoria Australia

**Keywords:** Delphi, podiatry, recommendations, sesamoiditis

## Abstract

**Introduction:**

Sesamoiditis is a common, and often painful, musculoskeletal pathology frequently encountered by podiatrists. However, there are currently no recommendations to guide podiatrists in the assessment and management of people with sesamoiditis. The aim of this study was to develop consensus‐driven clinical recommendations on the assessment and management of people with sesamoiditis.

**Methods:**

A four‐round online Delphi survey was conducted with a panel of New Zealand and Australian podiatrists. In the first round, panellists answered open‐ended questions that were used to create statements. In round two, the panellists scored the statements from 1 to 9 (1 = not at all important, 9 = absolutely essential). Consensus was defined using the RAND/University of California Los Angles Disagreement Index. Panellists were asked to reconsider statements that did not achieve consensus in round three. In the final round, content validity and acceptability of the statements for inclusion in clinical recommendations were determined using content validity ratios and the Content Validity Index (CVI).

**Results:**

Eighteen panellists completed round one with 16 (89%) completing all four rounds. A total of 118 statements were generated following round one. Following rounds two and three, 78 statements were accepted by panellists as being important, with 62 statements achieving sufficient content validity for inclusion in clinical recommendations. The CVI for these 62 statements was 0.58. These recommendations provide guidance on subjective assessment (pain characteristics/symptomology, activity/sports/training history and medical history) objective assessment (establishing a diagnosis, identifying contributing biomechanical factors, footwear/orthoses, ruling out differential diagnoses) and management (temporary padding/strapping, education, footwear, foot orthoses and when to consider referral).

**Conclusion:**

This consensus exercise has provided a set of consensus‐based recommendations for the assessment and management of people with sesamoiditis. In the current absence of research‐based evidence in this area, these recommendations are intended to support clinicians. The recommendations may also serve as a basis for future clinical trials evaluating the efficacy of conservative interventions for people with sesamoiditis.

## 1 INTRODUCTION

Sesamoiditis is a painful, musculoskeletal pathology involving acute or chronic inflammation of the sesamoid bones and/or surrounding soft tissue located at the plantar aspect of the first metatarsophalangeal joint [1, 2]. Although there are no data on the prevalence of sesamoiditis in the general population, the 4‐year incidence of sesamoiditis in athletes has been reported to be 4.2% [3], with sesamoiditis accounting for 18.3% of all first metatarsophalangeal joint injuries [4]. Activities involving repetitive loading or forced dorsiflexion of the first metatarsophalangeal joint, such as running and jumping, are reported to contribute to the development of sesamoiditis [5, 6, 7]. The clinical presentation typically involves a sudden or gradual onset of local or diffuse pain with difficulty in weightbearing on the first metatarsophalangeal joint [8]. As a result, people with sesamoiditis experience functional limitations and apropulsive gait strategies, which can be particularly challenging among active populations [1, 2].

Although sesamoiditis is one of the most common forefoot injuries encountered by podiatrists [4], there are currently no recommendations to assist podiatrists in their approach to the assessment and management of people with sesamoiditis. In response to the lack of published data on sesamoiditis [9, 10], a recent qualitative study involving focus group discussions between experienced podiatrists in Aotearoa New Zealand [11] found that podiatrists are guided by advanced clinical experiences and anatomical knowledge of the lower limb when caring for people with sesamoiditis. Their approach to assessing patients with sesamoiditis included both subjective questioning and a range of objective examinations focused on diagnosis and identification of contributing biomechanical factors. The treatment approaches described also varied considerably between podiatrists and was guided by their personal preferences and the patient's social factors, symptomology and assessment findings. Although a range of assessment and management techniques used by podiatrists were discussed in this study, further research is required to reach consensus and develop clinical recommendations. The Delphi process is a common strategy for reaching consensus, particularly when evidence is limited [12]. Therefore, the aim of this study was to develop consensus‐based clinical recommendations for podiatrists to use in the assessment and management of people with sesamoiditis.

## 2 METHODS

### 2.1 Study design

A four‐round Delphi technique was used to reach group consensus on assessment and management approaches for sesamoiditis [13]. The study design was informed by the Conducting and REporting of DElphi Studies recommendations [14]. Delphi studies are widely used in health research to determine the extent to which a group of expert panellists can reach consensus on a given issue. The results from Delphi studies are intended to guide health policy, clinical practice and research. Delphi studies overcome many of the disadvantages found with decision making in groups and committees by allowing panellist anonymity, and the ability of panellists to change their opinions as the Delphi rounds progress and in response to controlled sharing of the distribution of the group's responses [12].

### 2.2 Panellists

Potential participants were invited as panellists if they met the following inclusion criteria: registered podiatrists in New Zealand and Australia with at least 5 years of experience in predominantly musculoskeletal podiatry. These inclusion criteria were selected to ensure that panellists could provide the required knowledge and expertise. Potential panellists were identified through professional podiatry networks including Podiatry New Zealand, Sports and Exercise Podiatry Australia and New Zealand Accredited Sports Podiatrists. Individuals known to the research team with an interest in musculoskeletal podiatry were also contacted to determine their interest in the study and potentially forward the study information to their colleagues who met the study criteria. There is no consensus on group size and what defines ‘small’ or ‘large’ in Delphi studies [15]. However, evidence suggests that panellist groups over 30 rarely produce improved results [16]. A sample size of 15 to 20 panellists was considered adequate to obtain useful results. Ethical approval was obtained from the Auckland University of Technology Ethics Committee (AUTEC 23/117). All panellists provided consent prior to proceeding with the round one survey. It was made clear that participation would involve completing a total of four online surveys approximately 1–2 months apart. Panellists were anonymised to each other during the Delphi process to ensure that their contribution reflected their own clinical opinion and expertise [17].

### 2.3 Delphi process

The four Delphi survey rounds were administered between June and December 2023 via the online survey platform Qualtrics (Qualtric Research Suite Provo; UT 2013). To provide meaningful Delphi results, it is important that panellists complete all survey rounds. Therefore, published recommendations for online Delphi surveys were followed to maximise and retain participation across the surveys [18]. For rounds two through four, this involved sending weekly email reminders to panellists. Reminders were personalised (i.e., included the panellist member's name) and sent by one of the study researchers. Reminder emails continued to thank panellists for their contribution to the study, encouraged them to complete each survey round and emphasised the importance of their views.

#### 2.3.1 Round one

Panel members were asked to complete a short demographic questionnaire at the start of round one, which included questions related to years in practice, qualifications, workplace setting and region/country of practice. The first round included open‐ended questions asking panellists to describe their approaches to assessing and managing of people with sesamoiditis. Questions related to assessment included asking participants to describe what subjective information (patient history) they felt was important to obtain from a person with suspected sesamoiditis, and what assessments they felt were important in establishing a diagnosis of sesamoiditis and/or ruling out differential diagnoses. Management‐related questions included asking participants to describe both the short‐ and long‐term strategies they felt were important in the management of a person with sesamoiditis and when they felt it was important to refer on for further assessment and/or management. Participants were encouraged to be as detailed as possible in their responses. The round one Delphi survey remained open for 6 weeks to allow for the recruitment of an adequate number of panel members. A list of Delphi statements was then compiled based on data generated from these open‐ended questions as well as data from a recent qualitative focus group study conducted by the research team [11]. Every assessment and management approach shared by panellist members was deemed useful and included as a Delphi statement, regardless of the number of panellists who shared it. Similar approaches were grouped into single statements. The development and formation of the Delphi statements required ongoing discussion and refinement by research team members. The final statements were grouped into two categories: assessment of sesamoiditis and management of sesamoiditis. The Delphi statements were then piloted with two experienced podiatrists who were not involved as panellists to ensure that all statements were clear and understandable prior to their use in the second round.

#### 2.3.2 Round two

In the second round, the Delphi statements were presented separately for assessment and then management using the following two instructions: ‘*Please indicate how important you feel each of the following approaches are in assessment of a patient with sesamoiditis*’; ‘*Please indicate how important you feel each of the following management approaches are in treating a patient with sesamoiditis*’. To improve readability, subheadings were used to present statements on similar aspects of assessment or management. In this round, panellists were asked to indicate how important they felt each statement was using a 9‐point rating scale where 1 = not at all important and 9 = absolutely essential. This rating scale was selected to allow effective application of the RAND/University of California Los Angles Disagreement Index (RAND/UCLA DI) in subsequent rounds (as described below) [19]. The round two Delphi survey remained open for 4 weeks and was administered to all panel members who completed the round one survey.

#### 2.3.3 Round three

Following round two, statement consensus was determined using a combination of median statement scores and the RAND/UCLA DI [19]. Median statement scores between seven and nine and RAND/UCLA DI < 1 were deemed as important, and statements with median scores between one and three and RAND/UCLA DI < 1 were deemed as not important. Median statement scores between four and six or RAND/UCLA DI > 1 were deemed to lack consensus and were taken back to the panellists for further consideration in round three. In round three, panellists were also provided with the group responses (median rating) as well as a reminder of their own responses from round two and were asked to rescore each statement in light of this information [20]. Statements that still lacked consensus following round three were excluded from the final round. The round three survey remained open for 4 weeks and was administered only to panellists who completed the round two survey.

#### 2.3.4 Round four

The fourth and final round involved the evaluation of content validity of the statements that were considered important in rounds two and three. To maximise participation, all panellists who completed the round one survey were invited to participate in this final round, regardless of whether or not they had completed rounds two and three. Due to the New Zealand and Australian summer holidays coinciding within the period of administration of this survey, the round four survey remained open for 6 weeks to allow panellists ample opportunity complete it. The panellists were asked to categorise all statements into the following categories: ‘important’, ‘useful, but not important’, and ‘not important’ for inclusion in a final set of recommendations. The Content Validity Ratio (CVR) was used to determine the content validity of each statement included in this round using the following formula: CVR = (n℮ − *N*/2)/(*N*/2); where n℮ = number of panel members who consider the statement to be important; and *N* = total number of panel members [21]. This formula produces values ranging from +1 (perfect agreement) to −1 (perfect disagreement). Statements that achieve positive CVRs (i.e., those greater than zero) indicate that more than 50% of panellists rate the statement as important. These statements were included in the final recommendations for the assessment and management of sesamoiditis. The Content Validity Index (CVI) of the included statements was also calculated as an overall measure of content validity (CVI = mean CVR of the included statements).

### 2.4 Statistical analysis

All statistical analyses were performed in Microsoft Excel (Microsoft Corporation, Redmond, Washington USA).

## 3 RESULTS

### 3.1 Characteristics of panellist members

A total of 22 participants accessed the round one survey. Of these, four did not provide consent and/or did not complete the survey, leaving 18 participants who agreed to participate and completed the survey questions. Demographic details of the 18 participants who completed round one are shown in Table 1.

**TABLE 1:** Characteristics of participants who completed round one (*n* = 18).

Gender, *n* (%)	
Female	5 (28%)
Male	13 (72%)
Age range, *n* (%)	
30–39 years	6 (33%)
40–49 years	7 (39%)
50–59 years	4 (22%)
60–69 years	1 (6%)
Country of practice, *n* (%)	
New Zealand	10 (56%)
Australia	8 (44%)
Years in podiatric practice, *n* (%)	
5–9 years	3 (17%)
10–14 years	3 (17%)
15–19 years	4 (22%)
20–24 years	2 (11%)
≥25 years	6 (33%)
Workplace setting, *n* (%)	
Private practice	13 (72%)
Private practice and university/academia	5 (28%)
Highest formal education, *n* (%)	
Undergraduate diploma	3 (17%)
Bachelor's degree	4 (22%)
Postgraduate certificate/diploma/bachelor's degree with honours	4 (22%)
Master's degree	4 (22%)
Doctoral degree	3 (17%)
Percentage (%) of work based in musculoskeletal/sports podiatry	
Mean (SD)	77.3 (23.9)
Number of patients with sesamoiditis treated in past 6 months	
Mean (SD)	8.9 (7.1)
Range	2–30

### 3.2 Delphi findings

**FIGURE 1:**
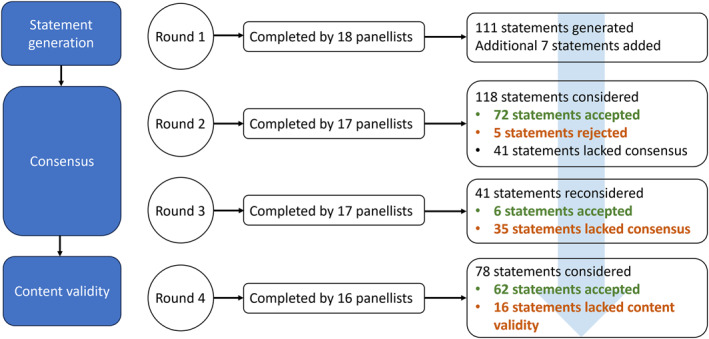
Delphi process.

Figure 1 outlines the Delphi process. A total of 111 statements were generated based on the responses from the 18 panellists who completed the round one survey. An additional seven statements were generated from the research team's recent focus group study [11], resulting in a total of 118 statements. These statements were organised by assessment (*n* = 56 statements) and management (*n* = 62 statements) approaches (see Supporting Information S1). Based on the open‐ended responses from the round one survey, it was also clear that the approach to assessment and management of sesamoiditis is tailored to each patient and not all approaches would be applicable to all patients. Of the 118 statements, 77 reached consensus in round two where 72 statements were accepted and 5 statements were rejected. The remaining 41 statements did not achieve consensus and were sent out to panellists for reconsideration in round three. Of the 41 statements reconsidered by panellists in round three, six statements were deemed important, while the remaining 35 statements were excluded due to lack of consensus. Of the initial 18 panellists, one did not complete survey rounds two and three. Statement consensus was therefore based on responses from 17 panellists. Table 2 presents the final 78 statements accepted by panellists as important to the assessment and management of sesamoiditis (72 were accepted in round two and six were accepted in round three). The content validity of these 78 statements was then assessed in round four. Of the initial 18 panellists, two did not complete round four. Therefore, content validity was determined based on the responses of 16 panel members. Of the 78 statements, 62 statements achieved positive CVRs (i.e., greater than zero), indicating that more than 50% of panellists rated the statement as important for inclusion in clinical recommendations (Table 2). The CVI of the included statements, which represents an overall measure of content validity, was 0.58.

**TABLE 2:** Statements accepted as being important in rounds two and three^a^ with their content validity ratio (CVR) from round four.

	Delphi round accepted	Median^b^ (IQR)	CVR^c^
Assessment			
Subjective assessment (patient history)			
To undertake a ‘PQRST’ pain assessment (including asking the patient questions regarding mechanism of injury/onset of pain, location of pain, quality/description of pain, pain severity [i.e. 0–10 rating scale], timing/pattern of pain, aggravating factors, relieving factors and duration of pain)	2	9 (8–9)	0.88
To determine whether the patient reports a history of swelling/bruising in the sesamoid area	2	7 (6–8)	0.63
To determine whether the patient has tried or received other treatment(s) for their current symptoms and, if so, the outcome of those treatment(s)	2	8 (8–9)	0.63
To determine whether the patient currently experiences pain in other regions of the foot or lower limb	2	8 (6–8)	−0.13
To determine whether the patient reports any difficulty/inability to walk barefoot due to pain	2	8 (7–8)	0.13
To determine whether the patient has a history of previous first metatarsophalangeal joint symptoms, injury, trauma or surgery	2	8 (7–9)	0.50
To determine whether the patient has a history of previous lower limb symptoms, injury, trauma or surgery	3	7 (6–8)	−0.25
To determine the patient's training load (including asking the patient questions around sport/position played, and frequency, intensity and duration of activity)	2	9 (9–9)	0.63
To determine which surfaces/terrain the patient trains on	2	8 (7–9)	0.38
To establish whether the patient has had any recent change to training/sports played	2	9 (8–9)	0.88
To determine the patient's occupation and lifestyle demands (including asking the patient questions around time spent sitting vs. weightbearing)	2	8 (8–9)	0.25
To determine what footwear the patient currently uses for sport, work and casual wear (including asking questions around any recent changes to footwear)	2	9 (8–9)	1.00
To determine the patient's expectations with treatment and any goals or objectives (including those related to sporting activities)	2	9 (8–9)	0.88
To take a history of any current systemic illness/comorbid condition that the patient has (including questions related to bone health, hormonal health, inflammatory disorders, chronic pain disorders, RED‐S and hypermobility disorders)	2	8 (7–9)	0.63
To determine whether the patient has received any recent abnormal blood test results	2	7 (6–8)	0.13
Objective assessment			
To palpate the medial and lateral sesamoids to recreate symptoms (with the hallux flexed and/or relaxed)	2	9 (9–9)	1.00
To assess for sesamoid translation/subluxation	2	7 (6–9)	0.25
To assess first metatarsophalangeal joint range of motion (including non‐weightbearing and/or weightbearing) for pain and/or restriction	2	9 (6–9)	0.38
To assess for the presence of any signs of inflammation localised to the area (including erythema, swelling and warmth)	2	9 (8–9)	0.75
To recreate the patient's symptoms through loading of sesamoids (including asking the patient to hop, walk on tip‐toes or do heel raises)	2	9 (8–9)	0.25
To perform gait analysis to assess for antalgic gait patterns including avoidance to propulsion	2	8 (7–9)	0.13
To assess the range of motion of the ankle, subtalar and midtarsal joints	2	8 (7–9)	0.75
To assess the strength of the hallux flexor muscles	2	8 (6–9)	0.38
To assess the strength of the hallux extensor muscles	3	7 (6–8)	−0.50
To assess strength of the fibularis/peroneus longus muscles	3	7 (6–9)	−0.13
To assess the strength of the ankle dorsiflexor and plantarflexor muscles	3	7 (7–9)	−0.13
To perform a gait analysis to determine the presence of any biomechanical factors contributing to first metatarsophalangeal joint overloading (including any asymmetries between limbs)	2	8 (7–9)	0.88
To assess first ray position and the range of motion	2	8 (8–9)	0.50
To assess the presence and severity of hallux valgus	2	7 (6–8)	0.00
To perform a Jack's test (including for assessment of the windlass mechanism)	2	8 (6–9)	0.25
To assess the presence/atrophy of the forefoot fat pad	2	7 (7–8)	0.25
To assess the patient's current footwear for fit, support, cushioning and age	2	9 (8–9)	0.75
To assess the patient's current footwear for insole wear patterns at first metatarsophalangeal joint	2	8 (8–9)	0.38
To assess the metatarsophalangeal joint flexion point in the patient's current footwear	2	7 (6–8)	0.25
To assess the patient's current orthoses (if applicable)	2	9 (8–9)	0.88
To palpate and assess adjacent soft tissue structures to rule out pathology (including pathology associated with the plantar plate, flexor tendons, joint capsule, collateral ligaments, interdigital neuroma and second metatarsophalangeal joint)	2	9 (9–9)	1.00
To refer for plain radiography (x‐ray) to rule out bone pathology (including assessment of bone integrity, bipartite sesamoid, fractures, avascular necrosis, crista/inter‐sesamoid pathology)	2	8 (7–8)	0.50
To refer for ultrasound imaging to assess pathology of soft tissue structures (including pathology related to bursa, joint capsule, plantar plate, ligaments and tendons)	2	7 (6–8)	−0.25
To refer for CT to rule out differential diagnoses (including when x‐ray or ultrasound may be inconclusive)	2	7 (5–8)	−0.38
To refer for MRI to rule out differential diagnoses (including when x‐ray or ultrasound may be inconclusive)	2	7 (6–8)	0.00
Management			
To apply initial temporary padding to the foot or shoe liner to offload the sesamoids (including the use of a U‐cut out/winged plantar cover, metatarsal dome and plantar metatarsal pad/T‐bar)	2	8 (7–9)	0.75
To apply strapping or taping to restrict/immobilise first metatarsophalangeal joint motion	2	8 (7–8)	0.13
To recommend the patient to wear footwear with an inbuilt forefoot rocker to facilitate sagittal plane movement	2	7 (7–8)	0.25
To recommend the patient to wear footwear with adequate space in toe box (depth and width)	2	7 (6–8)	0.13
To recommend the patient to wear highly cushioned footwear (particularly in forefoot)	2	7 (6–8)	0.38
To remove/reduce cleats/studs/sprigs in the sesamoid area (i.e. on football/hockey/rugby boots)	2	8 (6–8)	0.50
To recommend the patient to wear footwear with a suitable means of fixation to the foot (i.e. laces) to avoid deformation of the digits	2	7 (6–8)	−0.25
To recommend the patient to wear footwear that fits adequately (including correct length and width)	2	8 (7–9)	0.75
To recommend the to patient avoid shoes with too much metatarsophalangeal joint flexion	2	7 (7–8)	−0.25
To recommend the patient to avoid the use of high heels	2	9 (8–9)	1.00
To recommend the patient to avoid walking barefoot	2	7 (6–9)	0.38
To put the patient in a moonboot or CamWalker if symptoms/presentation are acute	2	8 (7–8)	0.38
To prescribe foot orthoses that aim to offload the sesamoids/first metatarsophalangeal joint loading (i.e. with first ray cut‐outs, plantar U covers, reverse Morton's extensions and metatarsal dome)	2	8 (7–9)	0.75
To prescribe foot orthoses that aim to provide more cushioning to the forefoot/sesamoid region (i.e. through the use of softer materials)	2	7 (6–8)	0.13
To prescribe foot orthoses that aim to address other contributing biomechanical factors (including controlling rearfoot motion)	2	7 (6–8)	0.38
To prescribe prefabricated foot orthoses with modifications	2	8 (7–8)	0.50
To prescribe prescription/customised foot orthoses	2	7 (6–8)	−0.25
To use a moonboot if symptom relief is not evident after a reasonable time frame	2	8 (7–9)	0.75
To provide reassurance and motivation to the patient	2	8 (8–9)	0.88
To provide the patient with education around the cause, management and prognosis of sesamoiditis	2	9 (8–9)	1.00
To recommend activity modification and/or rest if activity/training is identified as a contributing factor	2	9 (9–9)	1.00
To educate the patient on self‐immobilisation of first metatarsophalangeal joint motion	3	7 (6–8)	−0.63
To recommend a gradual return to loading/activity as tolerated	2	9 (8–9)	1.00
To recommend strengthening of the hallux/toe flexor muscles	2	7 (6–8)	0.25
To recommend strengthening of tibialis posterior muscle	3	7 (5–8)	0.00
To recommend strengthening of intrinsic foot muscles	2	7 (6–8)	−0.13
To address contributing systemic factors (including through referrals)	2	8 (7–9)	0.38
Reasons for referral			
When the patient is not responding to conservative management after a reasonable time frame	2	8 (7–9)	1.00
When the patient presents with an extremely acute presentation involving inability to bear weight	2	9 (8–9)	0.63
When the patient presents with extremely severe pain and an absence of any history of trauma	2	9 (8–9)	0.88
When there is evidence of contributing systemic illnesses/comorbidities	2	9 (8–9)	0.88
When there is a suspected or confirmed bone stress injury, fracture or avascular necrosis	2	9 (9–9)	0.88
When the patient is a paediatric patient	2	7 (6–8)	−0.25
When the patient is an elite sports person or athlete requiring a more rapid return to sport	2	8 (7–9)	0.00
When a cortisone injection may be needed	2	8 (6–9)	0.88
When the patient presents with a long duration of chronic symptoms	2	8 (7–9)	0.25
When the diagnosis remains uncertain based on other assessments (including podiatric assessments and imaging)	2	9 (8–9)	0.75
When the patient presents with RED‐S factors	2	9 (9–9)	1.00

^a^ Median statement scores between seven and nine and RAND/UCLA DI <1 were be deemed to reach consensus as being important.

^b^ Median rating from 1 = not at all important to 9 = absolutely essential.

^c^ Statements achieving positive CVRs (i.e., those greater than zero) indicate more than 50% of panellists rate the statement as important and are shaded green; statements lacking content validity are shaded red. Statements shaded green are included in the final recommendations.

### 3.3 Recommendations for the assessment and management of sesamoiditis

#### 3.3.1 Assessment of sesamoiditis

**FIGURE 2:**
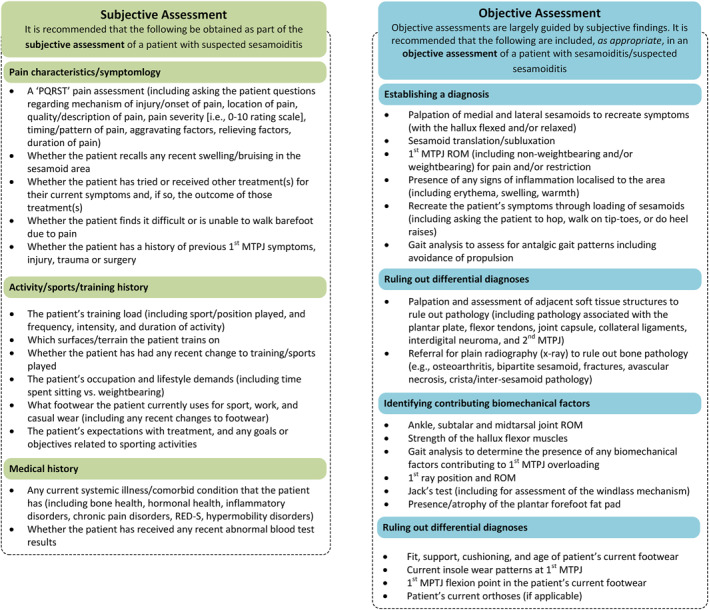
Recommendations for the assessment of a person with sesamoiditis/suspected sesamoiditis. 1MTPJ, first metatarsophalangeal joint; PQRST, pain assessment pneumonic; RED‐S, Relative Energy Deficiency in Sport; ROM, range of motion.

The Delphi process resulted in a total of 31 statements that were considered important for inclusion in clinical recommendations for the assessment of sesamoiditis. Recommendations are divided into two categories: subjective assessment (including pain characteristics/symptomology, activity/sports/training history and medical history) and objective assessment (including establishing a diagnosis, identifying contributing biomechanical factors, footwear/orthoses and ruling out differential diagnoses). Final recommendations for the assessment of a person with suspected sesamoiditis are presented in Figure 2.

#### 3.3.2 Management of sesamoiditis

**FIGURE 3:**
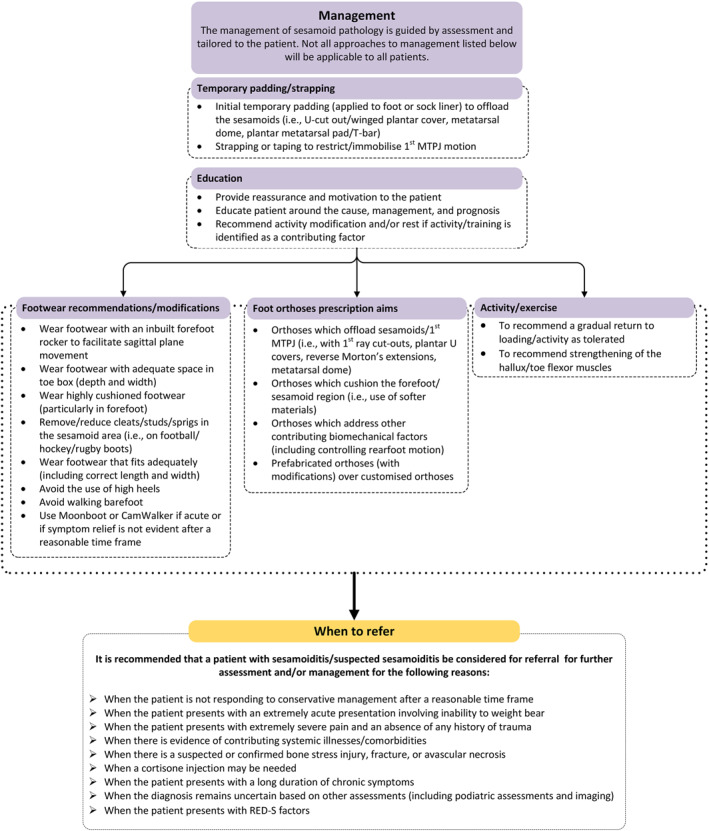
Recommendations for the conservative management of a person with sesamoiditis. 1MTPJ, first metatarsophalangeal joint; RED‐S, Relative Energy Deficiency in Sport.

A total of 31 statements were considered important to include in clinical recommendations for the management of sesamoiditis. Final recommendations for the management of a person with sesamoiditis including when to refer for further assessment and/or management are presented in Figure 3.

## 4 DISCUSSION

This Delphi study gathered the expertise from a panel of experienced podiatrists from Australia and New Zealand to develop consensus‐driven recommendations for the assessment and management of people with sesamoiditis. In the absence of research‐based evidence in this area, these recommendations will serve as a useful guide for podiatrists and direct future clinical trials aimed at determining the efficacy of podiatry‐led conservative management strategies specifically for people with sesamoiditis.

Anatomically, the sesamoid apparatus is a complex structure and the term sesamoiditis is used to describe many aspects of the pathology ranging from inflammation of the sesamoid bones themselves to inflammation of the supporting capsuloligamentous structures [1, 2]. These pathologies may include translocation or dislocation of the hallux or sesamoid bones, hyperextension injuries damaging the capsuloligamentous complex, plantar plate avulsions and sesamoid bone fractures [10, 22, 23]. The assessment approaches included in the final recommendations emphasise the importance of a thorough understanding of pain characteristics and symptomology, particularly in the presence of a complex anatomical structure. A comprehensive assessment of the patient's activity and training history was also included in the final recommendations, emphasising the strong connection between this pathology and sports activities in which the sesamoids are subjected to high forces and repetitive loads [9, 10]. In fact, dancing, running and football have been identified as the most common activities associated with the development of sesamoid bone injuries, accounting for 60% of all hallux sesamoid injuries [9]. Many of the recommended objective assessments also focus on identifying causes and consequences of sesamoid overload, including gaining an understanding of how biomechanical and footwear factors have contributed to the development of the condition. However, as the results of many of these objective assessments are interpreted subjectively, further research is required to establish their reliability and validity in assessing and diagnosing a person with suspected sesamoiditis.

Due to the complexity of the structure and overlap in clinical presentation, it may be clinically difficult to isolate the pathology to a specific musculoskeletal component of the sesamoid apparatus. Although advanced imaging techniques, including ultrasound, Magnetic Resonance Imaging and Computed Tomography can play an important role in narrowing down the location and cause of inflammation [24], these advanced imaging techniques were not considered to be important to include in the assessment of a person with sesamoiditis. Only plain radiography was included in the final recommendations as a measure to rule out potential bony pathology. This may indicate that practitioners feel confident in clinically assessing soft tissue pathology without the use of advanced imaging and/or believe that distinguishing between other causes of sesamoiditis aside from sesamoid fractures will not substantially alter their approach to management, particularly when there is limited research‐based evidence supporting one intervention over another for specific causes of sesamoiditis. The increased costs associated with imaging overuse, both to the healthcare system and patient, along with delays in diagnosis and therefore management, may also play a role in practitioners lack of advanced imaging referrals for sesamoiditis [25, 26]. Differences in referral pathways to more advanced imaging were notable between Australian and New Zealand panellists. The overall perceived low importance of advancing imaging in the assessment of sesamoiditis suggests that access to advanced imaging rights does not necessitate its use in patients with sesamoiditis.

Although a range of management options were recommended, in the absence of research‐based evidence the application of these recommendations should be based on clinical judgement and characteristics of the presenting individual. Following the use of temporary padding and education, footwear and orthoses were key components within the final recommendations. Contrary to evidence, which suggests that altering kinematics is effective at reducing pain [27], both footwear and orthotic strategies primarily focused on offloading and cushioning the sesamoids as opposed to encouraging a more efficient propulsive strategy through the sesamoid apparatus. A focus on offloading through padding and orthoses may therefore be the preferred method for people with sesamoiditis as opposed to altering joint biomechanics. However, clinical trials are needed to determine the effectiveness of these interventions.

Panellists also agreed on several reasons when referral for further assessment and management of person with sesamoiditis may be necessary. Some presentations of sesamoiditis may be beyond the scope of conservative podiatric treatment, particularly when the patient does not respond to conservative measures within a reasonable time frame, when the underlying cause may be due to systemic disease, or when treatment strategies are beyond the scope of the podiatrist's practice. Previous qualitative work has shown that podiatrists are hesitant to directly refer people with sesamoiditis for more invasive therapies due to concerns about low success rates and ongoing complications [11]. In fact, surgical management of sesamoiditis is not strongly evidence‐based [28] and is associated with a number of complications, including the development of hallux valgus, hallux varus, hallux extensus, claw‐toe deformity and weakness of hallux propulsion and gait changes [5, 29, 30].

Importantly, the recommendations developed from this study reflect the perspectives of each panel member, which, in the absence of research‐based evidence on the assessment and management of sesamoiditis, was influenced primarily by their own expert opinion and clinical experience. In addition, many of the approaches on which consensus has been reached are consistent with the principles used in the assessment and management of many other musculoskeletal disorders affecting the lower limb. This may also reflect the lack of high‐quality clinical trials evaluating the effectiveness of different assessment and management approaches specifically for people with sesamoiditis. Future clinical trials involving participants with sesamoiditis will likely influence future expert opinions and approaches to this pathology. Nevertheless, the current recommendations provide a basis for further evaluation and offer podiatrists with a consensus‐driven approach to assessing and managing a person with sesamoiditis.

This study had a number of strengths and limitations. Firstly, the panellists represented a wide range of clinical and academic experiences, which facilitated the exchange of views from both practical and theoretical perspectives. Secondly, the rigorous approach to maximising and retaining participation resulted in a high retention rate (89%) across the four Delphi rounds, ensuring that the findings adequately reflected the views of the available expert panel [18]. However, participation was limited to podiatrists practicing in Australia and New Zealand, where the majority work in private practice. The recommendations developed from this study may therefore not be as readily translatable in other countries or podiatry practice in secondary care or hospital settings. Panellists in this study were also limited to podiatric clinicians and future studies examining the experiences and opinions of other health professionals involved in the care of people with musculoskeletal lower limb problems may result in additional recommendations that could be applicable to wider healthcare services. Finally, the recommendations developed from this study were based on clinician's experiences and views and may not reflect the patient's opinion or preference. Further research could examine the patient experience of different management approaches, which is essential for developing strategies consistent with patient‐centred care.

In conclusion, the results from this Delphi study have enabled the development of consensus‐driven recommendations for the conservative assessment and management of people with sesamoiditis. With the current absence of research‐based evidence in this area, these recommendations may be helpful in assisting podiatrists in their approach to diagnosis, assessment and management of patients with this pathology. The recommendations may also serve as a basis for future clinical trials evaluating the efficacy of conservative interventions specific to people with sesamoiditis.

## AUTHOR CONTRIBUTIONS

**Sarah Stewart**: Conceptualization; data curation; formal analysis; methodology; project administration; writing – original draft preparation. **Preeti Kaur**: Conceptualization; resources; writing – review & editing. **Peta Tehan**: Methodology; resources; writing – review & editing. **Prue Molyneux**: Methodology; resources; writing – review & editing. **Matthew Carroll**: Conceptualization; formal analysis; methodology; visualization; writing – review & editing.

## CONFLICT OF INTEREST STATEMENT

SS, PT and MC are on the Editorial Board for the Journal of Foot and Ankle Research.

## ETHICS STATEMENT

Ethical approval was obtained from the Auckland University of Technology Ethics Committee (AUTEC 23/117). All panellists provided consent to participate.

## Supporting information

Supporting Information S1

## Data Availability

The data that support the findings of this study are available on request from the corresponding author. The data are not publicly available due to privacy or ethical restrictions.
