# Supplementary material for: The development of recommendations for the assessment and management of sesamoiditis by podiatrists: A Delphi and content validity study
Source: J Foot Ankle Res. 2024 May 31;17(2):e12025. doi: 10.1002/jfa2.12025 (PMC11296722; doi:10.1002/jfa2.12025)
Supplement: Supplementary file 1 — Supporting Information S1 [file JFA2-17-e12025-s001.docx]

| **Supporting Information.** 118 statements generated following round one |
| --- |
| **Statements related to the assessment of sesamoiditis (n=56)** |
| 1. To undertake a ‘PQRST’ pain assessment (including asking the patient questions regarding mechanism of injury/onset of pain, location of pain, quality/description of pain, pain severity [i.e., 0-10 rating scale], timing/pattern of pain, aggravating factors, relieving factors, duration of pain) |
| 1. To determine whether the patient reports a history of swelling/bruising in the sesamoid area |
| 1. To determine whether the patient has tried or received other treatment(s) for their current symptoms and, if so, the outcome of those treatment(s) |
| 1. To determine whether the patient currently experiences pain in other regions of the foot or lower limb |
| 1. To determine whether the patient reports any difficulty/inability to walk barefoot due to pain |
| 1. To determine whether the patient has a history of previous first metatarsophalangeal joint symptoms, injury, trauma or surgery |
| 1. To determine whether the patient has a history of previous lower limb symptoms, injury, trauma or surgery |
| 1. To determine whether the patient has a history of previous upper limb symptoms, injury, trauma or surgery |
| 1. To determine the patient’s training load (including asking the patient questions around sport/position played, and frequency, intensity, and duration of activity) |
| 1. To determine which surfaces/terrain the patient trains on |
| 1. To establish whether the patient has had any recent change to training/sports played |
| 1. To determine the patient’s occupation and lifestyle demands (including asking the patient questions around time spent sitting vs. weightbearing) |
| 1. To determine what footwear the patient currently uses for sport, work, and casual wear (including asking questions around any recent changes to footwear) |
| 1. To determine the patient’s expectations with treatment, and any goals or objectives (including those related to sporting activities) |
| 1. To take a history of any current systemic illness/comorbid condition that the patient has (including questions related to bone health, hormonal health, inflammatory disorders, chronic pain disorders, RED-S, hypermobility disorders) |
| 1. To determine whether the patient has a family history of systemic illness/comorbid conditions |
| 1. To determine the patient’s medication history (including questions related to current use of medications and medications recently ceased) |
| 1. To determine whether the patient has received any recent abnormal blood test results |
| 1. To determine whether the patient has experienced any recent weight gain |
| 1. To determine whether the patient is a current or previous smoker |
| 1. To determine whether the patient has any current allergies |
| 1. To palpate the medial and lateral sesamoids to recreate symptoms (with the hallux flexed and/or relaxed) |
| 1. To assess for sesamoid translation/subluxation |
| 1. To assess first metatarsophalangeal joint range of motion (including non-weightbearing and/or weightbearing) for pain and/or restriction |
| 1. To assess for the presence of any signs of inflammation localised to the area (including erythema, swelling, warmth) |
| 1. To recreate the patient’s symptoms through loading of sesamoids (including asking the patient to hop, walk on tip-toes, or do heel raises) |
| 1. To perform gait analysis to assess for antalgic gait patterns including avoidance to propulsion |
| 1. To assess range of motion of the ankle, subtalar and midtarsal joints |
| 1. To assess strength of the hallux flexor muscles |
| 1. To assess strength of the hallux extensor muscles |
| 1. To assess strength of the hallux abductor and adductor muscles |
| 1. To assess strength of the fibularis/peroneus longus muscles |
| 1. To assess strength of the ankle dorsiflexor and plantarflexor muscles |
| 1. To perform a gait analysis to determine the presence of any biomechanical factors contributing to first metatarsophalangeal joint overloading (including any asymmetries between limbs) |
| 1. To perform a plantar pressure analysis to assess first metatarsophalangeal joint loading patterns |
| 1. To assess for leg length discrepancy |
| 1. To assess the metatarsal parabola |
| 1. To assess first ray position and range of motion |
| 1. To assess the presence and severity of hallux valgus |
| 1. To assess static foot posture and arch height |
| 1. To perform a Jack’s test (including for assessment of the windlass mechanism) |
| 1. To assess the forefoot to rearfoot position and relationship |
| 1. To perform the supination resistance test |
| 1. To assess presence/atrophy of the forefoot fat pad |
| 1. To perform neurological testing (i.e., slump test, heel walk, straight leg raise) |
| 1. To assess the patient’s current footwear for fit, support, cushioning, and age |
| 1. To assess the patient’s current footwear for insole wear patterns at first metatarsophalangeal joint |
| 1. To assess the metatarsophalangeal joint flexion point in the patient’s current footwear |
| 1. To assess the patient’s current orthoses (if applicable) |
| 1. To palpate and assess adjacent soft tissue structures to rule out pathology (including pathology associated with the plantar plate, flexor tendons, joint capsule, collateral ligaments, interdigital neuroma, and second metatarsophalangeal joint) |
| 1. To use a percussion/tuning fork to rule out stress fracture |
| 1. To refer for plain radiography (x-ray) to rule out bone pathology (including assessment of bone integrity, bipartite sesamoid, fractures, avascular necrosis, crista/inter-sesamoid pathology) |
| 1. To refer for ultrasound imaging to assess pathology of soft tissue structures (including pathology related to bursa, joint capsule, plantar plate, ligaments, and tendons) |
| 1. To refer for CT to rule out differential diagnoses (including when x-ray or ultrasound may be inconclusive) |
| 1. To refer for MRI to rule out differential diagnoses (including when x-ray or ultrasound may be inconclusive) |
| 1. To refer for DEXA scan for assessment of bone density |
| **Statements related to the management of sesamoiditis (n=62)** |
| 1. To recommend/prescribe anti-inflammatory/pain relief medication (topical or oral) |
| 1. To recommend icing if acute or painful |
| 1. To apply initial temporary padding to the foot or shoe liner to offload the sesamoids (including the use of a U-cut out/winged plantar cover, metatarsal dome, plantar metatarsal pad/T-bar) |
| 1. To apply strapping or taping to restrict/immobilise first metatarsophalangeal joint motion |
| 1. To apply low-dye taping to reduce overpronation |
| 1. To apply a false plantar fascia taping to support medial longitudinal arch |
| 1. To recommend the patient wear footwear with an inbuilt forefoot rocker to facilitate sagittal plane movement |
| 1. To recommend use of a spring plate/carbon plate/gait plate in the patient's footwear |
| 1. To recommend the patient wear footwear with adequate space in toe box (depth and width) |
| 1. To recommend the patient wear highly cushioned footwear (particularly in forefoot) |
| 1. To add a full length poron insole to the patient's footwear to increase plantar cushioning |
| 1. To remove/reduce cleats/studs/sprigs in the sesamoid area (i.e., on football/hockey/rugby boots) |
| 1. To recommend the patient wear footwear with a steeper toe ramp/high pitch toe box |
| 1. To recommend the patient wear footwear with a suitable means of fixation to the foot (i.e., laces) to avoid deformation of the digits |
| 1. To recommend the patient wear footwear that fits adequately (including correct length and width) |
| 1. To recommend the patient avoid shoes with too much metatarsophalangeal joint flexion |
| 1. To recommend the patient use shoes that allow flexion at the level of the metatarsophalangeal joints |
| 1. To recommend the patient avoid the use of high heels |
| 1. To recommend the patient avoid walking barefoot |
| 1. To put the patient in a Moonboot or CamWalker if symptoms/presentation are acute |
| 1. To recommend the patient use crutches if acute |
| 1. To prescribe foot orthoses that aim to offload the sesamoids/first metatarsophalangeal joint loading (i.e., with first ray cut-outs, plantar U covers, reverse Morton’s extensions, metatarsal dome) |
| 1. To prescribe foot orthoses that aim to immobilise/limit first metatarsophalangeal joint motion (i.e., Morton’s extension) |
| 1. To prescribe foot orthoses that aim to provide more cushioning to the forefoot/sesamoid region (i.e., through the use of softer materials) |
| 1. To prescribe foot orthoses that aim to provide more control to foot function (i.e., through the use of firmer density materials) |
| 1. To prescribe foot orthoses that aim to facilitate more efficient first metatarsophalangeal joint motion |
| 1. To prescribe foot orthoses that aim to address other contributing biomechanical factors (including controlling rearfoot motion) |
| 1. To prescribe unmodified off-the-shelf prefabricated foot orthoses |
| 1. To prescribe prefabricated foot orthoses with modifications |
| 1. To prescribe prescription/customised foot orthoses |
| 1. To use a moonboot if symptom relief is not evident after a reasonable time frame |
| 1. To provide reassurance and motivation to the patient |
| 1. To provide the patient with education around the cause, management, and prognosis of sesamoiditis |
| 1. To recommend activity modification and/or rest if activity/training is identified as a contributing factor |
| 1. To educate the patient on self-immobilisation of first metatarsophalangeal joint motion |
| 1. To educate the patient on performing soft tissue massage of the medial longitudinal arch |
| 1. To perform laser therapy |
| 1. To perform acupuncture |
| 1. To undertake sesamoid glide manipulation |
| 1. To use prolotherapy (injection of glucose/lignocaine) |
| 1. To recommend a gradual return to loading/activity as tolerated |
| 1. To educate the patient on gait retraining if the gait pattern was identified as a contributing factor |
| 1. To recommend strengthening of the hallux/toe flexor muscles |
| 1. To recommend strengthening of intrinsic foot muscles |
| 1. To recommend strengthening of tibialis posterior muscle |
| 1. To recommend strengthening of fibularis longus muscle |
| 1. To recommend strengthening of proximal lower limb muscles |
| 1. To address contributing systemic factors (including through referrals) |
| 1. When the patient is not responding to conservative management after a reasonable time frame |
| 1. When the patient presents with an extremely acute presentation involving inability to weight bear |
| 1. When the patient presents with extremely severe pain and an absence of any history of trauma |
| 1. When there is evidence of contributing systemic illnesses/comorbidities |
| 1. When there is a suspected or confirmed bone stress injury, fracture, or avascular necrosis |
| 1. When the patient is a paediatric patient |
| 1. When the patient is an elite sports person or athlete requiring a more rapid return to sport |
| 1. When a cortisone injection may be needed |
| 1. When the patient presents with proximal factors influencing posture and gait |
| 1. When there is a suspected or confirmed ligament or tendon pathology |
| 1. When the patient fails to follow advice (i.e., rest) |
| 1. When the patient presents with a long duration of chronic symptoms |
| 1. When the diagnosis remains uncertain based on other assessments (including podiatric assessments and imaging) |
| 1. When the patient presents with RED-S factors |
